# Supplementary material for: Perfluorooctanesulfonate Can Cause Negative Bias in Creatinine Measurement in Hemodialysis Patients Using Polysulfone Dialysis Membranes
Source: Membranes (Basel). 2022 Aug 13;12(8):778. doi: 10.3390/membranes12080778 (PMC9413667; doi:10.3390/membranes12080778)

membranes

Supplement

Appendix A. Creatinine measurement

1. **The IDLCMS method** is determined as following steps: A 10-µl aliquot of each serum sample was diluted with 740 µl of distilled water and fortified with 500 µl of 1 µg/ml creatinine-D3 solution (C/D/N Isotopes Inc., Quebec, Canada). Three volumes of methanol were added to the above mixture to precipitate out proteins. After vortexing for one minute and centrifugation at 12,000×g for 10 minutes at 4oC, 500 µl of the supernatant were collected for further analysis. The quantitative LC-MS/MS analysis of creatinine was performed on an Agilent 1200 series SL RRLC system coupled to an Agilent 6410 triple quadruple mass spectrometer equipped with an ESI source in positive mode (Agilent Tech., Santa Clara, CA). One µl of the sample was injected onto a 2.1 mm × 100 mm Kinetex 2.6 µm HILIC column (Phenomenex Inc., Torrance, CA) with a mobile phase consisted of 0.1% formic acid in water (A) and in acetonitrile (B) at a constant flow rate of 0.3 ml /min. The following linear gradient program for elution was applied: 0-1 min held on 90% B, 1-5 min linearly decreased from 90% to 40% B, and held for one minute before returning to initial condition. Optimized MS parameters were as follows: capillary voltage of 4,000 V, drying gas temperature of 350°C, drying gas flow rate of 8 l/min, nitrogen nebulizer pressure of 30 psi, and dwell time of 200 ms. The detection was carried out by the multiple reaction monitoring (MRM) modes. The fragmentor voltage (V), collision energy (V), and MRM transitions monitored were as follows: 102, 15, m/z 117→47 for creatinine-D3; 100, 20, m/z 114→44 for creatinine. The monitored precursor and product ions were acquired by the Agilent MassHunter Qualitative software and quantified with the Agilent MassHunter Quantitative software. The standard at concentrations ranging from 0.0025 to 0.05 mg/dl was used to establish the standard curve constructed by plotting the ratio of peak area for creatinine to that for creatinine-D3 versus analyte concentration for six concentrations (0.0025, 0.005, 0.0075, 0.01, 0.025, and 0.05 mg/dl).

2. **Jaffe's colorimetric method**

This method is made by injecting patient's serum into an alkaline picrate solution. There are two reagents used in this method with components and concentrations listed as following: Reagent 1(R1) : Sodium hydroxide 0.2 mol/L, Reagent 2 (R2) : Picric acid 20 mmol/L. Mix 4 parts of R1 and 1 part of R2 to form a mono-reagent. The stability of mono-reagent: 5 hours at 15 – 25 °C. We incubated 50 ul of distill water with 1000 ul monoreagent for 60 seconds as blank and 50 ul of patient serum with 1000 ul monoreagent for 180 seconds as sample. Creatinine in the serum sample combines with alkaline picrate to form a red-colored complex or chromophore, the light absorbance of which can then be measured in the 490–510 nanometer range. The rate of absorbance is directly proportional to the creatinine concentration in the serum as compare to the blank.

3. **Enzymatic method:** it is determined by Beckman AU640 with amidohydrolase procedure which uses the reaction sequence:

Creatinine + H2O creatininase creatine

Creatine + H2O creatinase sarcosine + urea

Sarcosine + O2 + H2O sarcosine oxidase formaldehyde + glycine+ H2O2

The hydrogen peroxide generated in the above reaction sequence can be measured spectrophotometrically using a Trinder's reaction acceptor, producing a quinoneimine with high molar absorptivity. [18]

Appendix B. Measurement of PFOA and PFOS

All blood samples were stored before analysis at −80℃. The method for analysis of PFCs is as following: The frozen serum were thawed at 4℃ and vortex mixed for 30 seconds to reach homogeneity. A serum sample of 50 μL in a polypropylene centrifuge tube was then vortexed with 50 μL of 1% formic acid (pH 2.4) for 30 seconds. Afterwards, 1μL of 10 μg/mL internal standard solution (^{13}C4-PFOA and ^{13}C4-PFOS, Wellington Laboratories Inc., Guelph, Ontario, Canada) and 40 μL of acetonitrile were added to each sample before further vortex. These samples were sonicated for 20 mins and centrifuged at 18,000×g for 20 min. These supernatant were collected and filtered through a 0.22 μm polyether sulfone syringe filter to a screw cap vial.

In this study, the LC-MS/MS system used was comprised an Agilent 1100 series system (Agilent Tech., Santa Clara, CA, USA), and coupled to a Finnigan TSQ Quantum Discovery Max spectrometer system (Thermo Electron Corporation, Breda, Netherlands). The electron spray ionization source was in negative ion mode. LC-MS/MS and isotope dilution were carried out simultaneously for quantification of PFC.

A sample of serum (5 μL) was injected onto a 2.0 mm × 150 mm Capcell Pak® 3 μm C18 column (Shiseido Co., Tokyo, Japan). The mobile phases consist of 10 mM ammonium acetate in water (A) and pure acetonitrile (B) which delivered at a constant flow rate of 0.2 mL/min. After the injection, the mobile phase was kept for 3 min at 30% B. Then the gradient of B was increased to 65% in 3 min. Afterwards, the gradient of B was gradually increased 100% in 5 mins to 100% B, where it was kept for 7 mins. The column was set at 30% B for 1.5 min.

Optimized mass spectrometry parameters are listed below: spray ion voltage 3,000 V, capillary temperature 210℃, sheath gas pressure 10 arbitrary units, auxiliary gas pressure 5 arbitrary units, ion sweep gas pressure 4 arbitrary units, collision gas pressure 1.0 mTorr, and dwell time 100 msec. The detection was carried out in selective reaction monitoring (SRM) mode. The collision energy (V) and SRM transitions monitored were as follows: 10 V, m/z 413→369 for PFOA; 12 V, m/z 417→372 for 13C4-PFOA; 40 V, m/z 499→80 for PFOS; 40V, m/z 503→80 for 13C4-PFOS. We used LC-MS/MS to quantify PFOA (m/z 413 →369), PFOS (m/z 499→80) as the main particles for PFOA and PFOS for further analysis. [19]

Appendix-Figure S1 the molecular structure of PFOA and PFOS

Appendix-Figure S2 the product ion scans of PFOA and PFOS

Appendix-Figure S1 the molecular structure of PFOA and PFOS

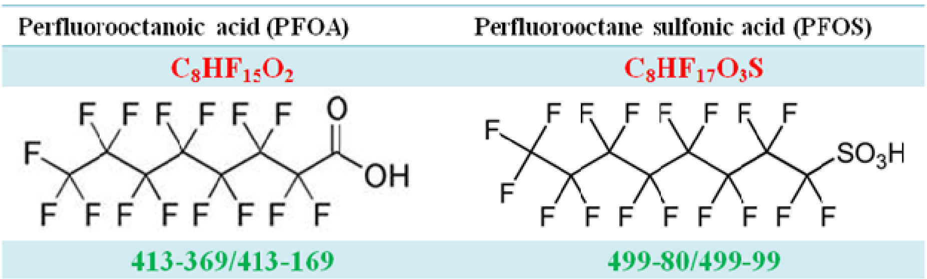

Appendix-Figure S2 the product ion scans of PFOA and PFOS

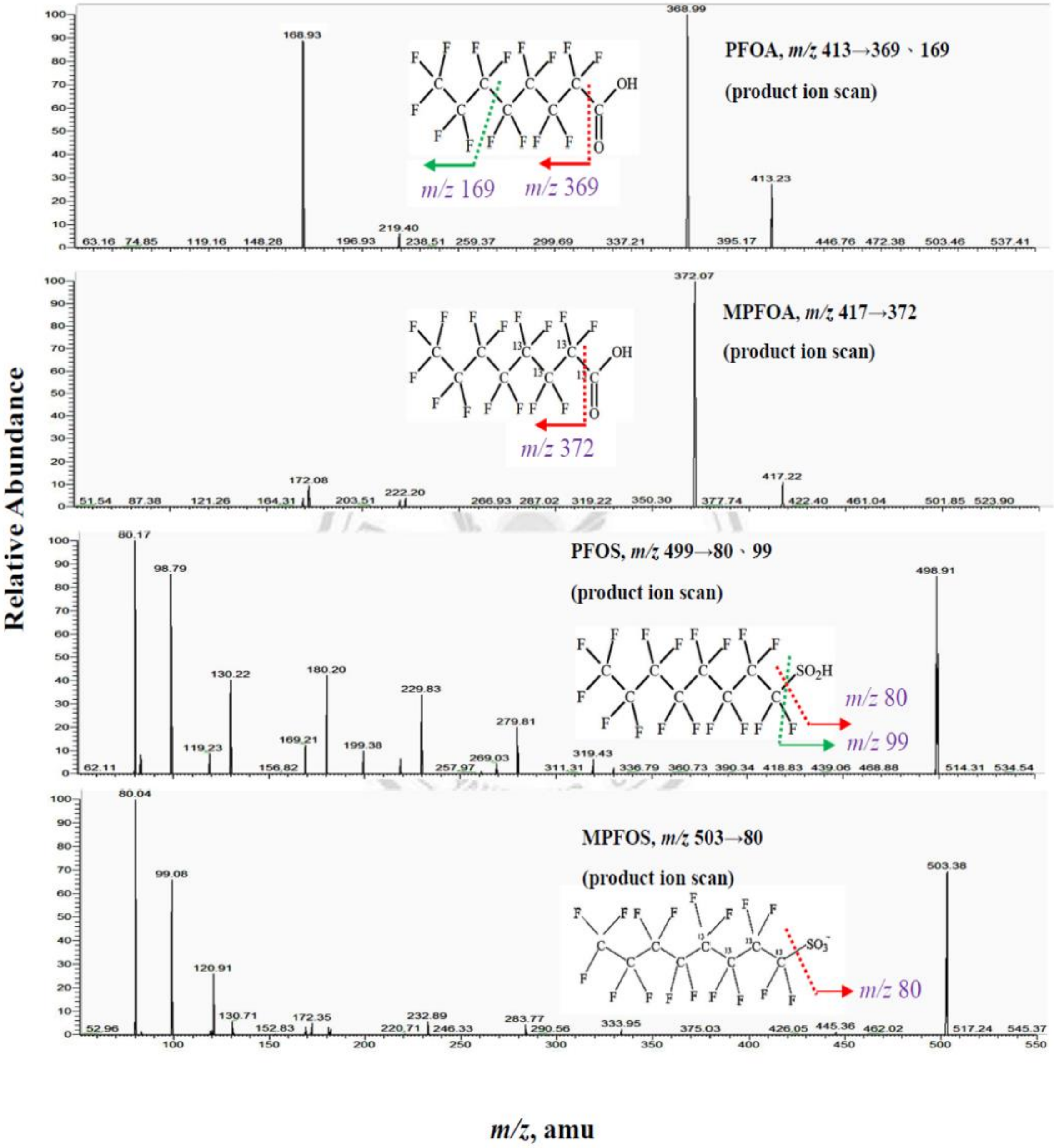

Supplement: Supplementary file 1 [file membranes-12-00778-s001.zip › membranes-1817583-supplementary.pdf]
